# Type of pre-existing chronic conditions and their associations with Merkel cell carcinoma (MCC) treatment: Prediction and interpretation using machine learning methods

**DOI:** 10.1371/journal.pone.0327964

**Published:** 2025-07-18

**Authors:** Yves Paul Vincent Mbous, Zasim Azhar Siddiqui, Murtuza Bharmal, Traci LeMasters, Joanna Kolodney, George A. Kelley, Khalid Kamal, Usha Sambamoorthi

**Affiliations:** 1 Department of Pharmaceutical Systems and Policy, School of Pharmacy, West Virginia University, Morgantown, West Virginia, USA; 2 AstraZeneca, Oncology, Outcomes Research, Boston, Massachusetts, United States of America; 3 OPEN Health, Evidence and Access, Real World Evidence, New York, United States of America; 4 Department of Hematology/Oncology, School of Medicine, West Virginia University, Morgantown, West Virginia, United States of America; 5 Department of Epidemiology and Biostatistics, School of Public Health, West Virginia University, Morgantown, West Virginia, United States of America; 6 School of Public and Population Health, Boise State University, Boise, IDIdaho, United States of America; 7 Department of Pharmacotherapy, College of Pharmacy, University of North Texas Health Sciences Center, Fort Worth, Texas, United States of America; Shuguang Hospital Affiliated to Shanghai University of Traditional Chinese Medicine, CHINA

## Abstract

**Objective:**

This study examined the prevalence of pre-existing chronic conditions and their association with the receipt of specific cancer-directed treatments among older adults with incident primary Merkel Cell Carcinoma (MCC) using novel predictive and interpretable machine learning methods.

**Methods:**

We adopted a retrospective cohort study design with data from linked Surveillance, Epidemiology, and End Results (SEER) registry and Medicare Fee-For-Service claims databases of older adults (≥ 66 years) diagnosed with primary incident MCC between 2008 and 2017. The study cohort consisted of 1,668 older adults with incident MCC and continuous fee-for-service Medicare enrollment for 24 months. Chronic conditions were identified during 12 months before cancer diagnosis date. Type of any MCC treatment (surgery-SRx, radiotherapy-RTx, chemotherapy-CTx, immunotherapy-ITx, and hormonal therapy-HTx) were derived for 12 months following cancer diagnosis. Receipt of any of these treatments and their associations with pre-existing chronic conditions were analyzed using separate eXtreme Gradient Boosting (XGBoost) predictive models and SHapley Additive exPlanations (SHAP) methods.

**Results:**

High cholesterol (75.5%), HIV (71.5%), hypertension (67.7%), arthritis (54.9%), coronary artery disease (47.1%), diabetes (43.5%), and hepatitis (37.1%) were some of the highly prevalent pre-existing chronic conditions. MCC treatment varied by type of chronic conditions and treatment modality. For example, a lower percentage of those with hypertension received ITx compared to those without hypertension (5.7% vs. 17.1%). A higher percentage of those with high cholesterol (13.9% vs 10.8%) received HTx compared to those without high cholesterol. XGBoost predictions revealed high predictive accuracy (area under the curve ranged from 0.72 (CTx) to 0.99 (ITx)). Hypertension (ITx), diabetes and thyroid disorders (HTx), congestive heart failure (RTx), and high cholesterol (CTx) were among the top ten predictors of MCC treatment. Congestive heart failure (RTx), hypertension (CTx), heart disease (ITx), thyroid disorders (HTx), and osteoporosis (HTx) positively predicted treatment, whereas high cholesterol (CTx), hypertension (ITx, HTx) and diabetes (ITx, HTx) negatively predicted treatment.

**Conclusions:**

Pre-existing conditions were highly prevalent among older MCC adults. Cardiovascular and metabolic diseases were the top 10 leading predictors of cancer treatment. However, the associations varied by type of treatment. In spite of the good performance of the model, especially for ITx and HTx, there is a need to replicate these findings using other data sources that provide access to larger population subgroups.

## Introduction

Merkel Cell Carcinoma (MCC) is a rare and aggressive cutaneous malignancy of neuroendocrine origin [1]. In the United States (US), the incidence of MCC has steadily increased since 1986 at a rate of 8% per year, increasing from 0.15 in 1986 to 0.44 per 100,000 population in 2001 [2]. However, post 2000, MCC incidence has increased by 95%, with rates going from 0.5 to 0.7 per 100,000 person-years in 2013 (2488 cases in total). A recent study showed that incidence rate of MCC cohort diagnosed between 2016 and 2021 was 1.0 per 100,000 persons-year, which corresponds to an adjusted incidence rate ratio of 1.54 compared to the reference (2001–2004 cohort) [3].

It is projected that incidence rates will reach more than 3248 cases per year by 2025 [4]. Older adults present higher risk of developing MCC. Between 2000–2013, incidence rates increased 10-fold between people aged 40–44 and 60–64 years and between those aged 60–64 and above 85 years [5]. Incidence cases among 60–64 years of age and those 85 years or above was 1.0 per 100,000 and 9.8 per 100,000, respectively.

Prior to 1970, surgery (SRx), radiation therapy (RTx), anticancer chemotherapy (CTx) and stem cell transplantation were the therapeutic modalities of choice in oncology. Between 1970 and 2023, the stage was set for the inclusion of hormone therapies and targeted therapies (photodynamic therapy, antibody drug conjugates, and immune checkpoint inhibitors) [6]. Historically, the standard of care in MCC has mimicked this treatment evolution [7]. In MCC, SRx is considered the mainstay of therapy across all stages, whereas evidence is inconclusive with respect to the best use of adjuvant RTx or CTx. The addition of targeted treatments such as immunotherapy (ITx) or hormonal therapy (HTx) is mostly directed towards metastatic MCC, and their benefits for those in other MCC stages have not been comprehensively assessed [8–10].

Individuals with cancer have high rates of chronic conditions, perhaps because they share common lifestyle risk factors including smoking, obesity, diet, physical inactivity and substance use [11]. The presence of chronic conditions before a diagnosis of cancer can play a critical role in receipt and type of cancer treatment [12]. Among older patients (aged 65 years or older), one in four survivors have at least one chronic condition, and 15% have two or more [13]. Cancer survivors with three or more chronic conditions have increased to 8.1 million in 2018 from 4.1 million in 2002 [11]. In this paper, we use the term “comorbidity”, because the coexistence of a medical condition in addition to a primary disease of interest (MCC in our case) is defined as a comorbidity [14]. Older adults with comorbidity are less likely to receive curative treatment than those without comorbidity [15].Various reports have highlighted the challenges of guideline-concordant management among comorbid cancer patients [16–19]. There are divergent opinions on the impact of comorbidity on treatment outcomes and effectiveness among cancer patients [15], however, there is clear indication of the adverse effect of comorbidity on survival [15].

Research studies on the prevalence of chronic conditions before cancer diagnosis have been limited to specific cancers (lung, prostate, and breast), and efforts to build evidence among other cancers have been limited [20]. To date, only one study described the top prevalent comorbidities in MCC, and it remains unclear whether these conditions were pre-existing or occurred post-diagnosis and treatment. Moreover, no study has actively investigated the association of pre-existing chronic conditions with receipt of MCC treatment. Understanding the association of comorbidities with MCC treatment type in real-world setting is important to support the care of patients with chronic conditions through program, policy, practice, and research. It is thus important to investigate this association across comorbidities that meet the definition of chronicity (conditions that last at least a year and necessitate medical intervention) and that are amenable to public health or clinical interventions [21].

Therefore, the main objective of this paper is to investigate the associations of specific comorbid conditions with receipt of different types of MCC treatment such as CTx, HTx, ITx, and RTx using linked and augmented database of a nationwide cancer registry, fee-for-service Medicare claims and geocoded databases among older adults with MCC. We used predictive and interpretable machine learning (ML) approaches to predict and assess the direction of these associations.

## Methods

### Study design

A retrospective cohort study of older adults (age ≥ 66 years at incident cancer diagnosis date) was conducted. Older adults with an incident primary diagnosis of MCC were identified using the International Classification of Diseases for Oncology, 3^rd^ edition histologic (ICD-O-3[8247]) and behavior (ICD-O-3 [3]) codes in the SEER registry. We used a baseline and follow-up period anchored to the incident cancer diagnosis date. The baseline period consisted of 12 months before cancer diagnosis and follow-up period consisted of 12 months following cancer diagnosis. All patient-level variables (features) were measured during the baseline period. Types of cancer treatment was derived from the follow up period.

### Data sources

The data for this study was derived from SEER-Medicare, which combines the following databases: 1) Surveillance, Epidemiology, and End Results (SEER) cancer registry; 2) Medicare enrollment; 3) fee-for-service Medicare claims. SEER data contains information pertaining to cancer site, stage, date of diagnosis (month, year), vital status, and cause of death on all incident cancers diagnosed among persons residing in the registry catchment areas, representing approximately 28% of the US population. Medicare enrollment data contain individual-level (older adults aged ≥ 65) demographic characteristics, enrollment information for Medicare Parts A, B, and D, Medicare Advantage (HMO), and dual Medicaid/Medicare coverages. Medicare claims data contains diagnosis, treatment, and payment information and include inpatient, outpatient hospital services, physician or supplier services, durable medical equipment, hospice, and home health care files. Part D prescription drug event data includes payments and dates of service for oral prescriptions drugs.

Cancer files from the SEER registry were linked to Medicare claims using encrypted patient IDs. This study was approved by the Institutional Review Board of West Virginia University (#2203549606). The linked SEER-Medicare data was accessed on a daily basis between November 08^th^, 2023 and January 7^th^, 2024 for this study purposes.

### Study period

**Fig 1:**
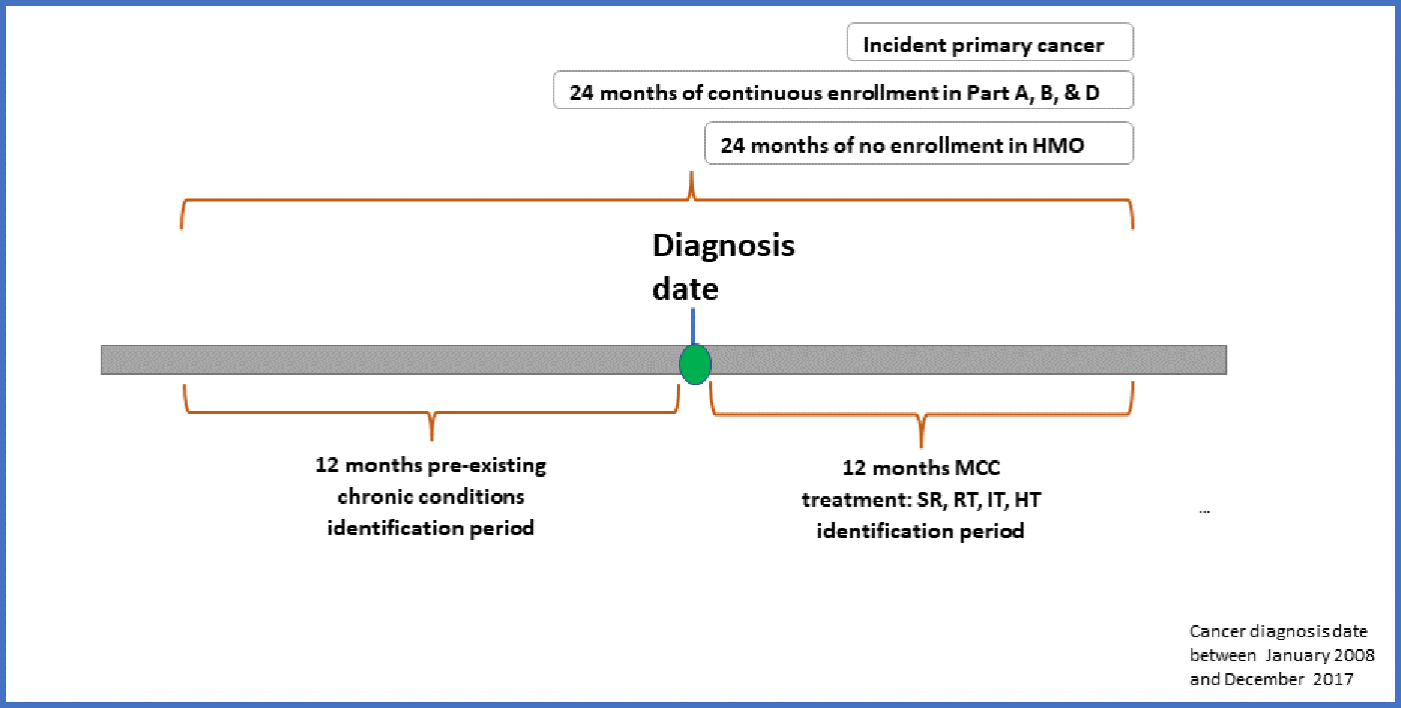
Study design: Linked SEER Cancer Registry and Medicare Claims files, 2008–2017.

The observation period spanned 13 years from 2007 through 2019 for incident MCC diagnosed between 2008 and 2017. We selected these periods because of availability of Medicare Part D in 2007. To accommodate 12 months baseline period before diagnosis of cancer, diagnosis years were restricted between 2008 and 2017 (Fig 1).

### Study cohort: Inclusion and exclusion criteria

**Fig 2:**
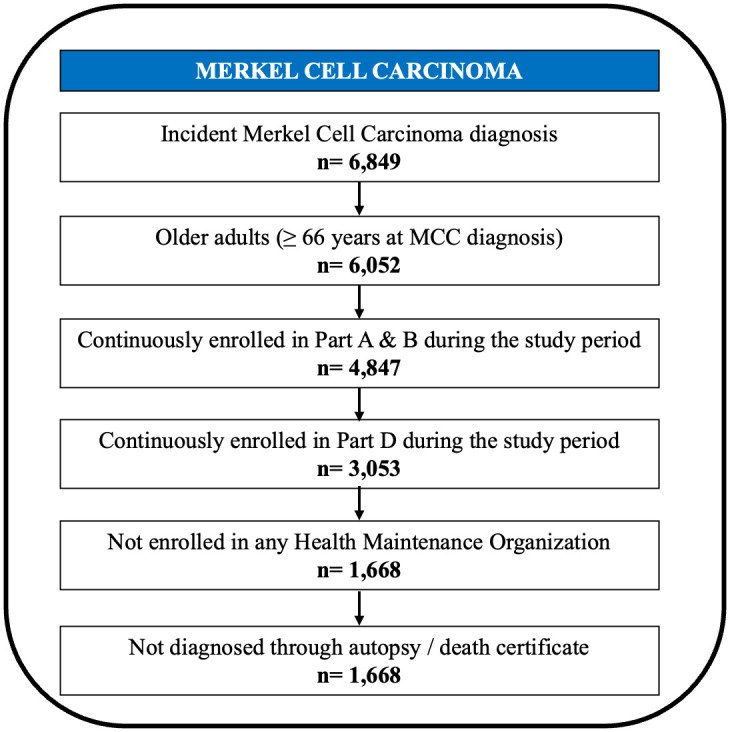
Inclusion and exclusion criteria: Older Adults Older Adults (age ≥ 66 years) with incident primary MCC Linked SEER Cancer Registry and Medicare Claims files, 2008 to 2017.

Medicare beneficiaries with diagnosed MCC were required to have: 1) a primary incident diagnosis of MCC; 2) aged 66 or older at the time of diagnosis; 3) continuous enrollment in fee-for-service Medicare with Parts A, B, and D during the baseline and follow up periods; 4) no HMO enrollment at any point during the baseline or follow up period. Patients with previous cancer diagnoses were excluded, as well as those diagnosed via death certificate or at autopsy. The final study cohort consisted of 1,668 older adults (age ≥ 66 years) with incident MCC between 2008 and 2017. The cohort selection is shown in Fig 2.

### Measures

### Target variables: Types of MCC treatment. 

The National Comprehensive Cancer Network (NCCN) guidelines v.2023 for MCC lists SRx, RTx, CTx, ITx, and HTx as mainstream therapeutic modalities for this cancer, however, ITx and HTx are relatively recent additions to this. Type of treatments were derived during the 12 months after incident MCC diagnosis and separate indicator variables (Yes/no) were created for each treatment (RTx, CTx, ITx, and HTx). The predictive model did not include SRx because an overwhelming majority (96%) of older adults received SRx. The type of treatment was identified from fee-for-service Medicare claims, specifically from outpatient, inpatient, home health agency, durable medical equipment, carrier, and prescription drug claims. We used the International Classification of Diseases, 9^th^, and 10^th^ edition Clinical Modification (ICD-9-CM, ICD-10-CM) and Procedure Codes (ICD-9-PCS, ICD-10-PCS), Healthcare Common Procedure Coding System (HCPCS), National Drug Codes (NDC), and Current Procedural Terminology (CPT) codes produced by the Centers for Medicare and Medicaid Services. These codes were collected from various sources: 1) SEER-Medicare online resources, 2) literature review; 3) the Healthcare Cost and Utilization Project (HCUP) repository; 4) and the online National Cancer Institute Observational Research in Oncology Toolbox: The Cancer Medications Enquiry Database (CanMED). To better capture all intakes of anti-neoplastic drugs, all the drugs listed in Part D claims were mapped to a list of antineoplastic drugs from CanMED database [22], and classified into CTx, HTx, and ITx. In addition to CanMED, we also used a list of cancer drugs from the National Cancer Institute (NCI) [23]. As the NCI list does not classify cancer drugs into CTx, ITx, HTx, two trained pharmacists reviewed the list to classify drugs into CTx, HTx, and ITx. The frequency of treatment type was calculated at baseline and after diagnosis. At baseline, it served as a checkpoint mechanism to ensure that we properly identified patients with no prior cancer related treatment. As these treatments can be administered as polytherapy, the most common being SRx and adjuvant RTx, we created a variable to multi-level variable accounting for the provision of the following: 1) SRx; 2) SRx and RTx; and 3) all other combination of treatment.

### Key features: Pre-existing chronic conditions

The presence of chronic conditions before cancer diagnosis was identified using ICD-9-CM and ICD-10-CM codes recorded in home health, carrier, MEDPAR, and outpatient claims. We then used the Clinical Classification Software (CCS mapped to ICD-9-CM) and the Clinical Classification Software Refined (CCSR mapped to ICD-10-CM) categorization schemes. These schemes -devised by the Agency for Healthcare Research and Quality (AHRQ) – allow mapping of a multitude of diagnosis or procedure codes into fewer relevant categories suitable for research. The CCS (ICD-9-CM) and CCSR (ID-10-CM) aggregate over 14,000 ICD-09-CM and 70,000 ICD-10-CM codes respectively into 284 and 530 clinical categories.

The list of chronic conditions identified before cancer diagnosis was based on the US Department of Health and Human Services (DHHS) strategic framework [21], otherwise known as the Goodman Framework. The following chronic conditions were included in this study: asthma, arthritis, congestive heart failure, coronary artery disease, chronic kidney disease, chronic obstructive pulmonary disease (COPD), cardiac dysrhythmias, diabetes, gout, heart failure, hypertension, HIV, hepatitis, high cholesterol, osteoporosis, stroke, thyroid disease, depression, and anxiety. Each of these conditions were categorized as binary variable.

### Other features/predictors

Additional features were selected based on published literature, relevance and meaning to healthcare stakeholders, and from elements available in the data. These features included race, biological, clinical, behavioral factors, social determinants of health (SDOH), and year of diagnosis. SDOH variables were based on the healthy people 2020 SDOH framework [24]. Biological factors included sex (female, male), and age in years at diagnosis. The race variable was categorized as White and Non-White due to the very small cell sizes (n < 11) observed for Black, Asian Pacific Islander, Hispanic, American Indian, or Alaska Native subgroups. Behavioral factors included alcohol, drug, trauma, suicide, and tobacco use identified using ICD-9-CM or ICD-10-CM codes. The SDOH included marital status (married, not married), the SEER region categorized as Northeast, South, Midwest, and West, insurance coverage (Medicaid dual insurance), pain specialist visits, psychologist visits, and fragmentation of care. The fragmentation of care index (FCI) was derived using a modified version of a previously validated continuity of care index [25]. The FCI requires three different inputs: the total number of healthcare encounters, the number of different providers, and the proportion of encounters with each of the providers. The FCI can take values from 0 (homogeneity of encounters, i.e., with the same provider) to 100 (heterogeneity of encounters, i.e., with different providers) with higher values indicating care discontinuation. For each interpretation, we divided the FCI by 100 so that each point represents a 10-percentage point increase in FCI, thus our FCI measure ranged from 0 to 1.

Clinical factors included cancer stage (localized MCC, regional MCC, and distant MCC) as identified through the SEER registry. Year of diagnosis was categorized as a continuous variable, "0" indicating 2008 and "9" indicating 2017.

### Analysis: Prediction

In this work, a decision-tree ensemble ML algorithm, Extreme Gradient Boosting (XGBoost), was selected as it offers several advantages [26]. Most notably, XGBoost allows the use of Shapley Additive exPlanations, a model-agnostic technique that facilitates the interpretability of XGBoost outputs [27]. Among other advantages, XGBoost: 1) combines multiple ML methods for improved predictive accuracy, 2) helps prevent overfitting through regularization and gradient boosting [26].

### Model-Agnostic interpretation

While ML models such as XGBoost have advantages, ML models in general are black-box models whose outputs are difficult to interpret [28]. To render XGBoost output explainable, we used a model-agnostic SHapley Additive exPlanations(SHAP), an interpretable ML technique that can translate the output into feature importance graphs, summary statistics, and visualizations to analyze feature importance, direction and associations of features with the target variables [27]. SHAP values have global and local interpretations. Globally, they explain positive or negative contribution of each feature to the dependent variable, whereas locally, they advise on by-patient contribution of features. As it is important to understand both how changing that feature impacts the model’s output, and the distribution of that feature’s values, we used SHAP dependency plots. The partial dependent plots (PDPs) can reveal the type of relationships (linear, monotonic, and more complex) of a feature with a target variable. We also used SHAP interaction plots to distinguish the contributions of features into their main and interaction effects. Xgbfir package was used to explain interactions and feature importance and partial dependence plots were generated using TreeSHAP in Python 3.11.4.

#### Data pre-processing.

As our linked databases are not ML ready, we engaged in data pre-processing steps. For example, one-hot encoding was used to transform categorical variables into numerical format suitable for ML [29]. The dataset was split into training and test sets, each respectively consisting of 70% and 30% of the dataset. Based on the conceptual framework, literature review and relevance to stakeholders, we selected 39 patient level variables.

#### Predictive model training and testing.

All ML analyses were conducted using Python 3.11.4. 10-fold cross validation were applied to the training set to ensure improved predictive accuracy for training and hold-out sets. In ML algorithms, specific parameters termed hyperparameters can be tuned to improve the learning process and performance. Tuned XGBoost hyperparameters included the following: maximum depth of a tree, gamma, colsample_bytree, learning rate, min_child_weight, n-estimators, reg_alpha, reg_lambda, scale_pos_weight, and subsample. Grid search was applied to identify the best performing hyperparameters values. Final model predictions were evaluated using the original hold-out test data.

#### Handling imbalanced data.

We faced imbalanced distributions in our binary variables for treatment with cases (Patients receiving treatment under examination) being less represented than controls across all treatment variables except RTx. When treatment and no treatment rates are not equal, it creates an imbalanced data, which is a challenge for classification methods. Most ML models for classification were developed for balanced data with an equal number of cases (i.e., treated) and controls (i.e., no treatment) [30]. Balanced classes are needed to improve model performance. Data-, algorithm-level, and hybrid approaches can be used to balance the data [31]. Data level approach involves random oversampling of cases and undersampling of controls. In our preliminary analysis, we observed that random over-sampling performed best in reducing class imbalance. Therefore, we applied random oversampling of cases (i.e., cancer treatment) to reduce class imbalance. We oversampled the final dataset to match a 1:2 ratio of cases-to-controls. For RTx, we applied a ratio of 1:1 of cases to controls because the cases were more predominant. These ratios were chosen based on the frequency required to remove samples from the minority while trying to generate new samples. The sampling strategy, a number between 0–1, is the desired ratio of the number of samples in the minority over those in the majority class after resampling.

#### Predictive model performance.

The performance of the models was evaluated using the test data set. We evaluated model performance based on accuracy (ratio of correctly predicted observation to the total observations), precision (ratio of correctly predicted positive observations to the total predicted positive observations), recall (sensitivity – ratio of correctly predicted positive observations to all observations in actual class), F1 score (weighted average of precision and recall), area under the receiver operating characteristic (AUC).

## Results

### Cohort characteristics

The study population (Table 1) was predominantly White (92.4%). Most beneficiaries were male (57.4%) resided in metro areas (92.6%) and 17.3% had dual Medicare/Medicaid coverage. The majority of older adults had tumors that were localized (64.1%) with unknown grade (89.7%).

**Table 1:** Selected sample characteristics among Fee-for-Service Medicare beneficiaries (age ≥ 66 years at index date) with MCC (n = 1,668), Linked SEER Cancer Registry and Medicare Claims files, 2008-2017.

Variable		N	Percentage (%)
**ALL**		1,668	100
***Biological determinants***			
***Sex***			
	*Male*	957	57.4
	*Female*	711	42.6
***Age***			
	*66-69*	180	10.8
	*70-74*	337	20.2
	*75-79*	339	20.3
	*80-84*	338	20.3
	*85,+*	474	28.4
***Race***			
	*White*	1,542	92.4
	*Non-White*	126	7.6
***Social determinants of health***			
***Marital status***			
	*Married*	804	48.2
	*Not Married*	864	51.8
***Dual Medicaid coverage***			
	*Yes*	288	17.3
	*No*	1,380	82.7
***Region***			
	*Northeast*	693	41.5
	*South*	277	16.6
	*Midwest*	154	9.2
	*West*	544	32.6
***Clinical variables***			
***Cancer stage***			
	*Local*	1,070	64.1
	*Regional*	401	24.0
	*Distant*	79	4.7
	*Unknown*	118	7.1
***Polypharmacy***			
	*Yes*	928	55.6
	*No*	740	44.4
***Chronic conditions***			
***Asthma***			
	*Yes*	150	9.0
	*No*	1,518	91.0
***Arthritis***			
	*Yes*	916	54.9
	*No*	752	45.1
***Congestive heart failure***			
	*Yes*	301	18.0
	*No*	1,367	82.0
***Coronary artery disease***			
	*Yes*	786	47.1
	*No*	882	52.9
***Cardiac dysrhythmias***			
	*Yes*	625	37.5
	*No*	1,043	62.5
***Chronic kidney disease***			
	*Yes*	285	17.1
	*No*	1,383	82.9
***COPD***			
	*Yes*	357	21.4
	*No*	1,311	78.6
***Diabetes***			
	*Yes*	725	43.5
	*No*	943	56.5
***Heart diseases***			
	*Yes*	405	24.3
	*No*	1,263	75.7
***Hypertension***			
	*Yes*	1,130	67.7
	*No*	538	32.3
***HIV***			
	*Yes*	1,193	71.5
	*No*	475	28.5
***Hepatitis***			
	*Yes*	619	37.1
	*No*	1,049	62.9
***High cholesterol***			
	*Yes*	1,259	75.5
	*No*	409	24.5
***Osteoporosis***			
	*Yes*	200	12.0
	*No*	1,468	88.0
***Stroke***			
	*Yes*	170	10.2
	*No*	1,498	89.8
***Thyroid diseases***			
	*Yes*	450	27.0
	*No*	1,218	73.0
***Depression***			
	*Yes*	231	13.8
	*No*	1,437	86.2
***Anxiety***			
	*Yes*	194	11.6
	*No*	1,474	88.4
***Lifestyle determinants***			
***Alcohol abuse***			
	*Yes*	27	1.6
	*No*	1,641	98.4
***Smoking***			
	*Yes*	30	1.8
	*No*	1,638	98.2
***Health care use***			
***Pain specialist visits***			
	*Yes*	459	27.5
	*No*	1,209	72.5
***Psychologist visits***			
	*Yes*	128	7.7
	*No*	1,540	92.3
***Emergency room use***			
	*Yes*	627	37.6
	*No*	1,041	62.4
***Continuous Variables***		**Mean**	**SD**
*Age at diagnosis (years)* *		79.39	7.68
*Care fragmentation index* *		0.75	0.15

Based on 1,668 older adults (age ≥ 66 years at index date) with MCC using data from Linked SEER Cancer Registry and Medicare Claims files during 2008–2017, who were continuously enrolled in Medicare Part A, and Part B during the study period, and Part D during the baseline period.

Care fragmentation = Bice-Boxerman continuity of care index to calculate care fragmentation during the 12-month baseline period (See Methods). Age: age at index date;

* Continuous variables for which mean and standard deviation is reported.

### Prevalence of pre-existing chronic conditions

In our study population, 98% had at least one chronic condition prior to MCC diagnosis. The prevalence rates of chronic conditions varied from as high as 75.5% (high cholesterol) to as low as 13.8% (depression). The highly prevalent conditions were HIV (71.5%), hypertension (67.7%), arthritis (55%) coronary artery disease (47.1%), diabetes (43.5%) and cardiac arrhythmias (37.5%).

### Type of cancer treatment

Among older MCC patients, RTx was the second most prevalent treatment (63.2%), after SRx (96.7%) (Table 2). CTx was administered among 31.2% of MCC beneficiaries, and HTx in 13.1%. ITx was prescribed to 9.4% of our patient population. In general, higher treatment rates were observed among those diagnosed with metastatic disease compared to local stage (RTx: 75.9% vs. 55.6%; CTx: 51.9% vs. 26.2%; ITx: 17.7% vs. 8.3%; HTx: 16.5% vs. 12.9%). We also analyzed combination therapy, which was common (43.2%).

**Table 2:** Type of pre-existing chronic conditions by treatment modality among Fee-for-Service Medicare beneficiaries (age ≥ 66 years at index date) with MCC (n = 1,668), Linked SEER Cancer Registry and Medicare Claims files, 2008-2017.

	Radiotherapy			Chemotherapy			Immunotherapy			Hormonal therapy		
	N	%	p-value	N	%	p-value	N	%	p-value	N	%	p-value
**Total**	1,054	63.2		520	31.2		156	9.4		219	13.1	
***Sex***			< 0.001			< 0.001			0.022			0.022
* Male*	643	67.2		340	35.5		103	10.8		110	11.5	
* Female*	411	57.8		180	25.3		53	7.5		109	15.3	
***Age***			< 0.001			0.005			0.680			0.900
* 66-69*	129	71.7		73	40.6		16	8.9		22	12.2	
* 70-74*	236	70.0		120	35.6		36	10.7		48	14.2	
* 75-79*	226	66.7		100	29.5		26	7.7		46	13.6	
* 80-84*	208	61.5		96	28.4		30	8.9		40	11.8	
* 85,+*	255	53.8		131	27.6		48	10.1		63	13.3	
***Asthma***			0.454			0.610			0.775			0.018
* Yes*	99	66.0		44	29.3		15	10.0		29	19.3	
* No*	955	62.9		476	31.4		141	9.3		190	12.5	
***Arthritis***			0.934			0.224			0.955			0.586
* Yes*	578	63.1		297	32.4		86	9.4		124	13.5	
* No*	476	63.3		223	29.7		70	9.3		95	12.6	
***Congestive heart failure***			0.444			0.021			0.853			0.780
* Yes*	196	65.1		77	25.6		29	9.6		41	13.6	
* No*	858	62.8		443	32.4		127	9.3		178	13.0	
***Coronary artery disease***			0.635			0.748			0.554			0.086
* Yes*	492	62.6		242	30.8		70	8.9		115	14.6	
* No*	562	63.7		278	31.5		86	9.8		104	11.8	
***Cardiac dysrhythmias***			0.405			0.161			0.343			0.299
* Yes*	387	61.9		182	29.1		53	8.5		89	14.2	
* No*	667	64.0		338	32.4		103	9.9		130	12.5	
***Chronic kidney disease***			0.695			0.033			0.455			0.510
* Yes*	183	64.2		104	36.5		30	10.5		34	11.9	
* No*	871	63.0		416	30.1		126	9.1		185	13.4	
***COPD***			0.959			0.970			0.119			0.279
* Yes*	226	63.3		111	31.1		41	11.5		53	14.8	
* No*	828	63.2		409	31.2		115	8.8		166	12.7	
***Diabetes***			0.749			0.336			0.007			0.136
* Yes*	455	62.8		217	29.9		52	7.2		85	11.7	
* No*	599	63.5		303	32.1		104	11.0		134	14.2	
***Heart diseases***			0.898			0.052			< 0.001			0.067
* Yes*	257	63.5		142	35.1		58	14.3		64	15.8	
* No*	797	63.1		378	29.9		98	7.8		155	12.3	
***Hypertension***			0.127			< 0.001			< 0.001			0.055
* Yes*	700	61.9		299	26.5		64	5.7		136	12.0	
* No*	354	65.8		221	41.1		92	17.1		83	15.4	
***HIV***			0.095			< 0.001			< 0.001			0.919
* Yes*	739	61.9		327	27.4		77	6.5		156	13.1	
* No*	315	66.3		193	40.6		79	16.6		63	13.3	
***Hepatitis***			0.242			0.006			< 0.001			0.478
* Yes*	380	61.4		168	27.1		38	6.1		86	13.9	
* No*	674	64.3		352	33.6		118	11.2		133	12.7	
***High cholesterol***			0.854			0.006			0.261			0.102
* Yes*	794	63.1		415	33.0		112	8.9		175	13.9	
* No*	260	63.6		105	25.7		44	10.8		44	10.8	
***Osteoporosis***			0.037			0.788			0.170			0.009
* Yes*	113	56.5		64	32.0		24	12.0		38	19.0	
* No*	941	64.1		456	31.1		132	9.0		181	12.3	
***Stroke***			0.363			0.221			0.173			0.687
* Yes*	102	60.0		46	27.1		11	6.5		24	14.1	
* No*	952	63.6		474	31.6		145	9.7		195	13.0	
***Thyroid diseases***			0.127			0.878			0.036			0.009
* Yes*	271	60.2		139	30.9		31	6.9		75	16.7	
* No*	783	64.3		381	31.3		125	10.3		144	11.8	
***Depression***			0.380			0.539			0.923			0.042
* Yes*	140	60.6		68	29.4		22	9.5		40	17.3	
* No*	914	63.6		452	31.5		134	9.3		179	12.5	
***Anxiety***			0.174			0.937			0.107			0.432
* Yes*	114	58.8		60	30.9		12	6.2		22	11.3	
* No*	940	63.8		460	31.2		144	9.8		197	13.4	

Almost all MCC patients with or without chronic conditions had surgery. Because the cell sizes were less than 11 for many characteristics, we do not present the results by surgery. The treatment for MCC varied by chronic conditions. For example, the percentage of MCC patients who received RTx ranged from a low 56.5% (osteoporosis) to 66% (asthma); CTx varied from 25.6% (congestive heart failure) to 36.5% (chronic kidney failure); ITx varied from 5.7% (hypertension) to 14.3% (heart diseases). HTx varied from 11.7% (diabetes) to 19.0% (asthma and osteoporosis).

### ML model performance (Test data)

**Fig 3:**
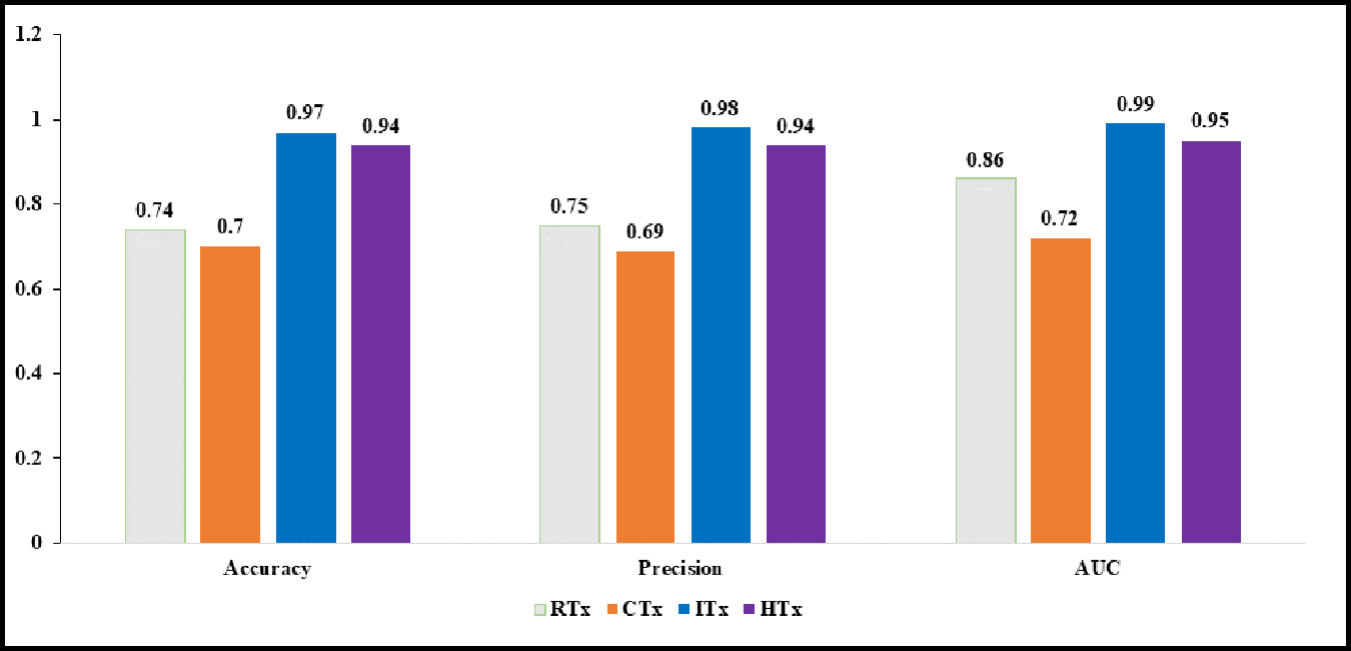
Performance Metrics of XGBoost Models:Older Adults Older Adults (age ≥ 66 years) with incident primary MCC Linked SEER Cancer Registry and Medicare Claims, 2007-2018. Legend: Based on 1,668 older adults (age ≥ 67 years diagnosis data) with MCC diagnosed between 2008-2017, who were continuously enrolled in Medicare Parts A, B & D during baseline period and follow up period. RTx: Radiation Therapy; CTx: Chemotherapy; HTx: Hormone therapy; ITx: Immunotherapy.

The model performance metrics assessed for each target variable using the test set are shown in Fig 3. All performance measures were high (> 0.90) for the target variables HTx and ITx. However, model performances were not as high for CTx and RTx. For example, AUC was 0.72 for CTx and 0.86 for RTx. Similar, patterns were observed for precision, recall, and accuracy.

### ML model interpretation: global feature importance of chronic conditions

**Fig 4:**
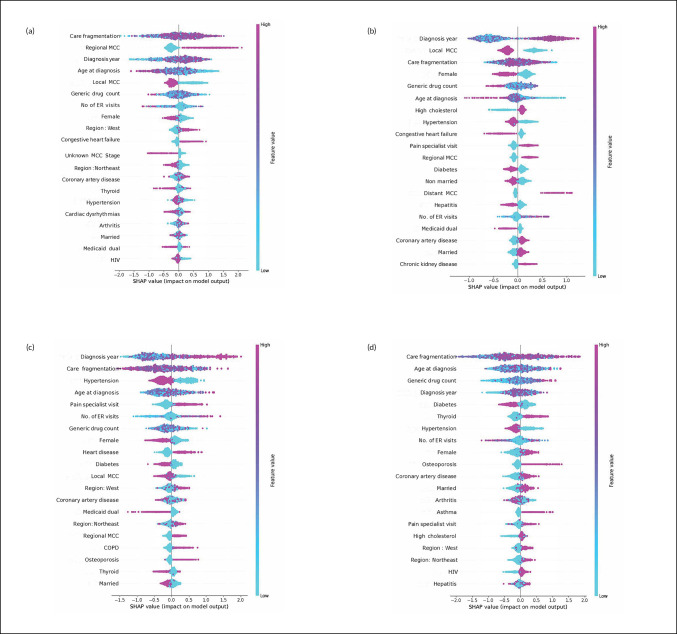
Shapley Additive exPlanations: summary plot with top 20 leading predictors Older Adults Older Adults (age ≥ 66 years) with Incident Primary MCC Linked SEER Cancer Registry and Medicare Claims files, 2007-2018. Legend: The x-axis represents the marginal contribution of a feature to the change in log odds of type of treatment. Based on 1,668 older adults (age ≥ 67 years diagnosis data) with MCC diagnosed between 2008-2017, who were continuously enrolled in Medicare Parts A, B & D during baseline period and follow up period; a) RTx; b) CTx; c) ITx; d) HTx. SEER- Surveillance, Epidemiology, and End Results Cancer Registry.

As shown in Fig 4 with the feature importance plot, a few chronic conditions such as congestive heart failure, diabetes, high cholesterol, hypertension, osteoporosis, and thyroid disorders were among the top 10 predictors of cancer treatments. For example, diabetes, thyroid disease, hypertension, and osteoporosis were respectively the 5^th^, 6^th^, 7^th,^ and 10^th^ leading predictors of HTx. High cholesterol, hypertension and congestive heart failure ranked 7^th^, 8^th^, and 9^th^ in prediction of CTx, whereas CHF was the 10^th^ leading predictor for the use of RTx. Hypertension, heart disease, and diabetes ranked as 3^rd^, 9^th^, and 10^th^ leading predictors of ITx.

### ML model interpretation: association of chronic conditions with type of treatment – SHAP summary plots

The SHAP summary plot in Fig 4 ranks features based on their predictive performance for the receipt of a particular therapy. It highlights feature contribution as well as the directionality of the relationship between features and the receipt of MCC treatment. Each dot at the feature level corresponds to an individual patient, and the thickness of the line is characterized by the number of people with similar characteristics. The colors vary according to feature values with pink representing large values and blue small values. SHAP values extending towards the negative side of the horizontal axis inform on a reduced chance of receiving a particular therapy and SHAP values extending towards the positive denotes a positive likelihood of receiving said therapy.

Diabetes was associated with a negative prediction of ITx and HTx. High cholesterol was associated with a positive prediction of CTx, whereas congestive heart failure was associated with a positive prediction of RTx.

### ML model interpretation: partial dependence plots of chronic conditions

**Fig 5:**
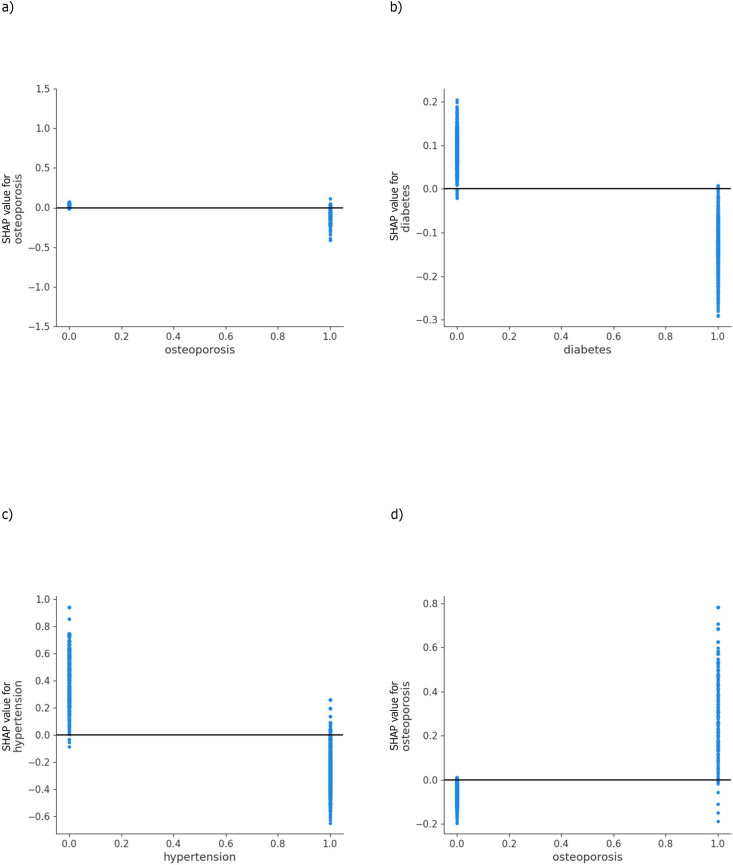
Shapley additive explanations (SHAP) dependence plots: Older Adults Older Adults (Age ≥ 66 Years) with Incident Primary MCC Linked SEER Cancer Registry and Medicare Claims files, 2007-2018. Legend: Each point on the plot corresponds to a prediction in a patient (a) & (b) SHAP dependence plot of log-odds SHAP values by care fragmentation and osteoporosis showing their main effect on receipt of RTx; (c) & (d) The main effect of arthritis and diagnosis year on receipt of CTx; (e) & (f) The main effect of care fragmentation and chronic kidney disease on receipt of ITx, and (g) & (h) The main effect of diagnosis year and diabetes on receipt of HTx. Based on 1,668 older adults (age ≥ 67 years at index date) with incident MCC diagnosed between 2008-2017, who were continuously enrolled in Medicare Part A, B and D during the baseline and follow up periods. SEER- Surveillance, Epidemiology, and End Results Cancer Registry.

As the chronic conditions were coded as binary indicators, the partial dependence plots reveal the distribution of the predictions for presence or absence of the condition. For example, those with osteoporosis were more likely to receive HTx and less likely to receive RTx (Fig 5); however, the positive predictions were heterogenous. Those with diabetes were less likely to receive CTx, and those with hypertension were less likely to receive ITx. The direction of association for the top 10 leading chronic conditions is also depicted in Table 3. Hypertension was negatively associated with ITx and HTx, whereas high cholesterol was positively associated with CTx.

**Table 3:** Key Feature interactions from XGBoost Classification Models Older Adults (Age ≥ 66 Years) with Incident Primary MCC Linked SEER Cancer Registry and Medicare Claims Files, 2008 to 2017.

Feature interactions	Gain	F1 Score	Weighted F1	Gain Rank	F1 Score Rank	Weighted F1 Score Rank
**Radiotherapy**						
Care fragmentation | Diabetes	127.9	48	1.5	26	24	67
Age at diagnosis |Heart diseases	119.9	47	2.9	28	25	34
Age at diagnosis | Diabetes	117.1	50	1.5	29	21	64
Diagnosis year | Hypertension	108.9	29	3.0	33	53	33
Diagnosis year | Thyroid diseases	107.0	37	4.5	35	37	23
**Chemotherapy**						
Diagnosis year | gout	90.4	5	1.9	13	68	29
Care fragmentation | Hypertension	57.6	5	1.5	19	69	44
Care fragmentation | Heart diseases	48.0	13	1.1	24	19	60
Distant MCC| Hypertension	45.1	4	2.3	25	78	24
Care fragmentation | Hepatitis	44.1	11	1.0	28	26	64
**Immunotherapy**						
Hepatitis | Hypertension	284.0	6	1.8	8	97	65
Age at diagnosis | Hypertension	244.0	8	2.0	10	90	53
Diagnosis year | Diabetes	198.6	17	2.6	14	67	38
Age at diagnosis | Coronary artery disease	185.8	44	3.6	17	17	30
Care fragmentation | Coronary artery disease	168.7	46	3.5	19	16	31
**Hormonal therapy**						
Care fragmentation | Asthma	137.6	21	5.8	16	60	19
Care fragmentation | Arthritis	131.4	46	3.5	17	17	30
Care fragmentation | Diabetes	129.0	50	6.6	18	15	15
Care fragmentation | Hypertension	125.9	42	4.8	19	21	22
Age at diagnosis | Coronary artery disease	123.4	35	4.5	20	24	23

### ML model interpretation: feature interactions with chronic conditions

Table 4 shows the top interactive associations of chronic conditions with patient-level features used in this study. For RTx, we observed that age at diagnosis interacted with heart disease, and diabetes at two distinct levels. For CTx, fragmentation of care interacted with hypertension, heart diseases, and hepatitis distinctly. For ITx, fragmentation of care interacted with coronary artery disease. For HTx, fragmentation of care interacted at four distinct levels with asthma, arthritis, diabetes, and hypertension.

**Table 4:** Associations of chronic conditions with cancer treatment among Fee-For-Service Medicare beneficiaries (age ≥ 66 years at incident cancer diagnosis) with MCC Linked SEER Cancer Registry and Medicare Claims Files, 2008 to 2017.

	*CTx*	*ITx*	*HTx*	*CTx*	*ITx*	*HTx*
Chronic conditions	Direction			Rank		
Diabetes		*+/-*	*+*		*10*	*5*
Hypertension	*+/-*	*–*	*–*	*11*	*3*	*7*
High cholesterol	*+*		*+/-*	*7*		
Heart diseases		*–*			*9*	
Osteoporosis	*+/-*		*+/-*	*13*		*10*
Thyroid diseases		*+/-*	*+*			*6*

Note: Blue: Mixed relationship; Green: positive relationship; Red: Negative relationship

**Fig 6:**
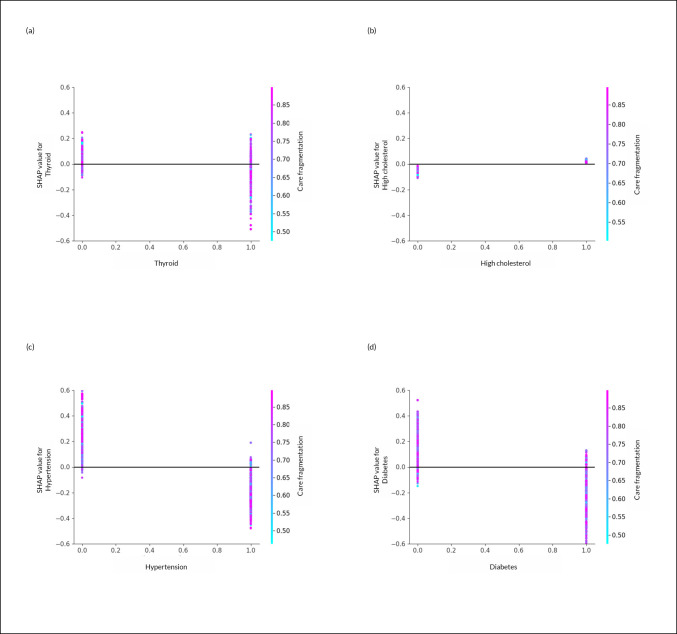
Shapley additive explanations (SHAP) interaction plots of the features: Older Adults Older Adults (Age ≥ 66 Years) with Incident Primary MCC Linked SEER Cancer Registry and Medicare Claims files, 2007-2018. Legend: Each point on the plot corresponds to a prediction in a patient (a) The SHAP interaction plot of thyroid diseases versus care fragmentation for the receipt of RTx. (b) The SHAP interaction plot of high cholesterol versus care fragmentation for the receipt of CTx. (c) The SHAP interaction plot of hypertension versus care fragmentation for the receipt of ITx and (d) The SHAP interaction plot of diabetes versus care fragmentation for the receipt of HTx. Based on 1,668 older adults (age ≥ 67 years at index date) with MCC using data from Linked SEER Cancer Registry and Medicare Claims files during 2008-2017, who were continuously enrolled in Medicare Part A, and Part B during the study period, and Part D during baseline period; Care fragmentation = Bice-Boxerman continuity of care index to calculate care fragmentation during the 12-month baseline period (See Methods). SEER- Surveillance, Epidemiology, and End Results Cancer Registry; NSAIDs-Non-steroidal anti-inflammatory drugs; SHAP-Shapley Additive exPlanations.

Fig 6 shows the interaction between some of the top chronic conditions and fragmentation of care and age at diagnosis. The relationship is highly mixed. For instance, for RTx, occurrence and non-occurrence of thyroid disease was associated with high levels of care fragmentation (Fig 6(a)). The same was observed for high cholesterol with CTx, hypertension with ITx, and diabetes with HTx (Fig 6).

### ML prediction, interpretation of other features

The common leading predictors across all treatments included fragmentation of care, MCC stage (local or regional), year of diagnosis, age at diagnosis, and generic drug count. The direction of association was highly mixed for these features. For instance, among the top predictors of RTx (fragmentation of care: rank = 1, year of diagnosis: rank = 3, age: rank = 4, generic drug count: rank = 6), the direction of association was highly complex. The PDPs of fragmented care, age at diagnosis revealed a complex relationship.

## Discussion

Pre-existing conditions were highly prevalent among older MCC Medicare beneficiaries, especially cardiovascular, metabolic, autoimmune, inflammatory, and infectious diseases. In our study, high cholesterol (75.5%), HIV (71.5%), hypertension (67.7%), and diabetes (43.5%) had the highest prevalences. The type and prevalence of pre-existing chronic conditions has rarely been investigated in MCC. A retrospective cohort study using electronic health records of MCC patients in Italy reported a prevalence rate of 27% for diabetes, much lower than the rate observed in our study [32]. A US led observational study of MCC patients using MarketScan claims found comparable rates of COPD (23.1%), higher rates of cerebrovascular disease (19.2%), and lower rates of congestive heart failure (14.2%), and renal disease (14.0%) at follow-up (post-diagnosis) [33]. Another work dedicated to metastatic MCC patients identified using the Premier Healthcare Database and treated with CTx in the US, uncovered anemia (34%), diabetes (34%), and thyroid diseases (18%) as the most predominant comorbidities [34]. Our study findings may not be comparable to the published studies as these studies did not distinguish between pre-existing chronic conditions or chronic conditions that developed after cancer diagnosis. Nevertheless, our findings suggest that MCC patients have to be assessed for pre-existing comorbidities and proper management of comorbidities undertaken, as the trajectory of cancer can vary according to its interaction with comorbidities, and thus resulting in excess mortality, risk, and disease progression than would be incurred by either acting independently [35,36].

MCC treatment rates found in our work are line with published literature. As found in our study, SRx is typically applied in more than 90% of all MCC cases [37,38]. RTx has been reported to range from 50% to 60% depending on the data source [38–40]. On the other hand, CTx use was higher in our study (31.2%) compared to literature findings (6.5% − 11%) across all stages [38,40,41]. The differences in CTx among studies can be possibly attributed to differences in populations with regard to cancer stage, source of data, and time period during which the treatments are captured. Until 2017, ITx and HTx were not common modes of treatment among MCC patients, as reflected in our study.

Among those with pre-existing conditions, a registry based-study found that RTx and CTx were administered in 100% and 29%, respectively in MCC patients with HIV, and in 95% and 37% in MCC patients with autoimmune diseases [42]. In this work, RTx and CTx were administered in 62% and 27.4% of MCC cases with pre-existing HIV. Among MCC patients with autoimmune disorders, the prevalences of RTx and CTx were 63.3% and 32.4%, respectively. Although our estimates are comparable to those in the literature, the slight discrepancies could be attributed to the methods used in identifying and stratifying the types of chronic conditions. Our approach was consonant with the DHHS framework used to distinguish chronic conditions, and is therefore very robust and rigorous. The use of the CCS and CCSR mapping in this study allowed us to capture vital and precise information on pre-existing chronic conditions. To the best of our knowledge, no past study on MCC that investigated, in some form, the presence of chronic conditions among MCC patients has ever used this approach.

Our study uncovered positive, negative as well as mixed relationships between pre-existing diagnosis and the receipt of MCC treatment. For example, those with pre-existing hypertension and diabetes were less likely to receive CTx or ITx or HTx, consistent with findings from a systematic review that reported that nonstandard and less aggressive treatments were administered preferentially to older cancer adults with comorbidities [12]. Another study showed that among breast cancer survivors, RTx and CTx were offered less in the presence of a high comorbidity burden [43]. In other cancers, it has been suggested that chronic conditions affect treatment, and are highly associated with the receipt of suboptimal care [15].

However, in our study, other chronic conditions such as high cholesterol, thyroid disorders and osteoporosis that were among the top predictors, were associated with higher likelihood of treatment. Although not related to MCC, a moderately positive and statistically significant associations between COPD, diabetes, and CTx were observed among colon cancer survivors [44]. However, a diagnosis of congestive heart failure was associated with higher (RTx) and lower (CTx) likelihood of MCC treatment. It is plausible that certain type of conditions may preclude the receipt of specific treatment. At times these disparities in treatment appear to be justified, however, the high preponderance of mixed relationships suggests a lack of rigor and high uncertainty with regards to decision making. As such, the highest quality patient-centered care remains unelucidated in many cases. Our study findings suggest that the relationship of comorbidities with cancer treatment among MCC patients is complicated in real-world practice settings. It is plausible that the lack of consensus on MCC treatment in the presence of chronic conditions may contribute to this complexity [45]. As clinical trials often exclude older adults with comorbidities owing to their frailty, information from real-world database studies can serve as a baseline benchmark for programs, policies, interventions, and practice to develop targeted and personalized treatments for MCC patients.

Although our findings point to an association between cardiovascular and metabolic comorbidities and the receipt of CTx, ITx and/or HTx, the large heterogeneity observed herein does not facilitate a global conclusion. These findings reinforce the need for a personalized approach to MCC management in the presence of comorbid conditions.

In this work, other features ranked top in prediction of the type of treatment. At the top featured fragmentation of care (RTx and ITx), and year of diagnosis (CTx and HTx). The relationship of care fragmentation with treatment type was highly heterogeneous, suggesting a complex interaction. Care fragmentation is widespread among cancer patients [46]. In 2013, the Institute of Medicine identified it as a priority area for the improvement of cancer care delivery and the reduction of healthcare costs [46]. Previous findings showed that fragmentation of care was more common among prostate cancer survivors who received RTx as their initial treatment [47]. This corroborates our study finding where for RTx, the top predictor was fragmentation of care. We speculate that care fragmentation may reflect appropriate need, and visits to different providers may lower the likelihood of receiving specific MCC cancer care; for others, it may reflect the fact that fragmented care may lead to a higher likelihood of receiving a specific MCC cancer treatment. Due to the complexity of their needs, comorbid cancer patients may benefit from seeing multiple specialists [48–50], and this may account for the lower probability of receiving cancer treatment at certain levels of care fragmentation. Future studies are needed to unpack the benefits of seeking care from multiple providers and the likelihood of receiving MCC optimal care.

With respect to year of diagnosis, we speculate that the advent of newer targeted therapies influenced their administration to MCC beneficiaries. For instance, ITx in the form of avelumab received accelerated approval in 2017, and since then has been a game changer for MCC. Further as a rare condition, diagnosis and staging was not accurately reported prior to 2009 [1]. The growing awareness of this condition has spurred interest on its improved care management, which could help explain why the year of diagnosis is a top predictor for receipt of CTx and HTx.

The model performance was moderate to high based on accuracy, precision, recall, F1 score, and AUROC values, suggesting the feasibility of ML models even in small size populations. The applicability of XGBoost needs to be replicated using other data sources; however, our results show that the use of predictive modeling is a useful tool in modeling cancer treatment when the number of variables is large and the population size is small [51].

This study holds many strengths. This study represents the first examination of the type and presence of pre-existing comorbidities in MCC using a large registry dataset linked with claims data. SEER-Medicare data, a dataset with a near-complete census information on all cancers in older adults (≥ 66 years) was used. The rigor in mapping chronic conditions is also an advantage as we used established categorization schemes for this purpose. A retrospective cohort design was used to establish and maintain the temporal relationship between pre-existing chronic conditions and type of treatment administered.

This study was limited mainly in the choice of features to use in our model. For example, we did not control for obesity, diet, patient preferences because of lack of information. Second, we did not include geographic level variables in our final model because multidisciplinary cancer care teams generally consider immediate clinical factors and patient level factors in their decision-making while neglecting geographical or costs factors [52]. Thus, we believed that this analysis would reflect the reality on the ground. Third, the use of ICD-9-CM and ICD-10-CM for the identification of chronic conditions in 2015 could be a potential limitation. In October 2015, the Centers for Medicaid and Medicare mandated providers covered under the Health Insurance Portability and Accountability Act (HIPAA) to transition from the ICD-9-CM system to the ICD-10-CM system [53]. The ICD-10-CM uses almost five-fold more codes than ICD-9-CM, and it is estimated that a third of ICD-9-CM codes do not have easily discernible corresponding ICD-10-CM codes [53]. Reports have mentioned significant changes in measured rates of injury-related hospitalizations, office -based physicians visits, identification of particular population subgroups (pregnant women), and the incidence of severe maternal morbidity as a result of this transition [54–57]. Thus, there is a possibility that measurement errors may have occured during the identification of chronic conditions during 2015, which required the use of both ICD-9-CM and ICD-10-CM systems. Fourth, while we captured a comprehensive list of chronic conditions from claims based on the DHHS strategic framework, claims data lack information specific to severity of chronic illnesses. However, published literature suggest that the number of chronic conditions may be a proxy for severity of illness [58].

Last, our findings may not be generalizable to the entire MCC population or Medicare population as our study included only older fee-for-service Medicare beneficiaries. The database lacked some key variables such as patient preferences, obesity, and other behavioral factors that may affect receipt of treatment. Furthermore, claims-based measures of depression, drugs, alcohol, and tobacco use have been known to have low sensitivity, as noted on the SEER-Medicare website.

## Conclusion

This study showed that pre-existing chronic conditions influence the receipt of cancer treatment among older Medicare beneficiaries with MCC. Although a significant number of chronic conditions were associated with treatment, there was a not a clear direction with respect to these conditions. Given the high prevalence of chronic conditions in older adults, future research is needed to identify the optimal treatment patterns to achieve the best outcomes for MCC patients with comorbidities.
